# Explore the potential mediating role of plasma metabolites and cytokines in the causal relationship between gut microbiota and the severity of immune-related adverse events: A Mendelian randomization study

**DOI:** 10.1097/MD.0000000000047916

**Published:** 2026-02-28

**Authors:** Sihan Liu, Jingtao Ma, Zhenli Li, Mengjia Li, Tenghui Wang, Tiezhu Yao, Guang Liu

**Affiliations:** a The Fourth Hospital of Hebei Medical University/The Tumor Hospital of Hebei Province, Shijiazhuang, Hebei Province, People’s Republic of China; b Department of Cardiology, The Fourth Hospital of Hebei Medical University/The Tumor Hospital of Hebei Province, Shijiazhuang, Hebei Province, People’s Republic of China; c Hebei Medical University, Shijiazhuang, Hebei Province, People’s Republic of China.

**Keywords:** cytokines, gut microbiota, ICI, irAEs, Mendelian randomization, metabolites

## Abstract

The gut microbiota, particularly genus *Ruminiclostridium6*, has been implicated in modulating immune-related adverse events (irAEs) associated with immune checkpoint inhibitor therapy. However, the mediating roles of circulating metabolites and cytokines in this relationship remain poorly understood. We performed a two-sample Mendelian randomization study to investigate causal links between genus *Ruminiclostridium6*, 19 plasma metabolites, 41 cytokines, and high-grade irAEs. Genetic instruments were selected from publicly available genome-wide association studies summary statistics. Inverse variance weighted method served as the primary analysis, supplemented by sensitivity and mediation analyses. No robust causal relationships were found between genus *Ruminiclostridium6* and the 19 metabolites. However, high-grade irAEs were causally linked to decreased oleoylcholine levels. Among cytokines, only IL-2RA showed a causal association with high-grade irAEs, but not with genus *Ruminiclostridium6*. Consequently, two-step Mendelian randomization did not support a mediating role for the studied metabolites or cytokines in the genus *Ruminiclostridium6*–irAEs pathway. Enrichment analysis highlighted glycine, serine, threonine, arginine, and proline metabolism as potential pathways of interest. This study did not support 19 plasma metabolites or 41 cytokines as major mediators of the protective effect of genus *Ruminiclostridium6* against high-grade irAEs. The mechanism may involve local gut-level immunomodulation or microbial metabolites not captured in this study. These findings underscore the complexity of microbiota–irAEs interactions and highlight specific metabolic pathways for further investigation.

## 1. Introduction

Immune checkpoint inhibitors (ICIs), which target cytotoxic T lymphocyte-associated protein-4, programmed cell death protein-1 (PD-1) or programmed cell death ligand-1 (PD-L1), have become a major treatment for cancer patients.^[1]^ By interrupting the inhibitory signaling pathways of T-cell inhibition, ICIs can reinvigorate the T cells to recognize tumor antigens and recover the antitumor immune response.^[2]^ However, ICIs can alter self-tolerance, and they are often associated with a spectrum of autoimmune-mediated toxicities that are collectively known as immune-related adverse events (irAEs).^[3]^ According to statistics, irAEs comprise over 70 different pathologies affecting nearly every organ system, including the skin, colon, lungs, heart, kidneys, and endocrine systems.^[4,5]^ IrAEs are common and have been reported to occur in 90% of patients treated with anti-cytotoxic T lymphocyte-associated protein-4 and 70% of patients treated with anti-PD-1/PD-L1.^[6]^ A recent systematic review of 125 clinical trials involving 20,128 patients showed an overall incidence of 66.0% of irAEs of all grades and 14.0% of grade 3 or above following treatment with PD-1 or PD-L1 inhibitors.^[7]^ More notably, the development of high-grade irAEs is significantly associated with a reduced overall survival in patients.^[8]^ Therefore, early identification and proactive management of high-grade irAEs represent an imperative clinical priority to reduce this negative impact on survival.

In recent years, several studies have increasingly focused on the core role of gut microbiota in the toxicity regulation of ICIs.^[9,10]^ For example, Liu et al identified a series of gut microbiota as causal risk or protective factors for irAEs.^[11]^ Especially, genus *Ruminiclostridium6* was identified as a reliable microbiota with a significantly protective effect for the high-grade irAEs, which was still significant when reevaluated by the methods in our study. However, the specific pathways by which the genus *Ruminiclostridium6* exerts its effects remain unclear and reverse causal relationships between the 2 traits are still need to elucidate. The metabolome delivers a highly dynamic and real-time representation of the body’s overall functional state. They serve not only as the terminal markers of gene environment interactions, but also as active molecules directly involved in immune regulation and other functional processes, such as short chain fatty acids, tryptophan derivatives, and amino acids.^[12,13]^ Furthermore, perturbations in metabolite levels, including elevated acylcarnitines and steroid hormone metabolites, are strongly related to different pathological processes in cancer treatment.^[14,15]^ Metabolomic profiling of peripheral blood has recently been reported to evaluate immune responses in cancers treated with ICIs.^[4,16,17]^ Recently, Tang et al^[18]^ revealed causal relationships from 22 metabolites with known names to the occurrence of high-grade irAEs by Mendelian randomization (MR), which highlights the important role of metabolites in the development of irAEs. However, they did not analyze the reverse relationships between the 2 traits. Moreover, 19 of 22 metabolites were still significantly associated with high-grade irAEs according to the rules in our study, which we utilized to process such reverse-relationship analysis. As for cytokines, several studies have explored its association with irRAEs. For example, Flatt et al^[19]^ elucidated IL-7, IL-1RA, and CXCL-13 enable early identification of patients at high risk for irAEs. In clinical practice, a case^[20]^ suggests that residual IL-6 suppression from prior tocilizumab therapy may attenuate the severity of subsequent irAEs. However, no MR study has ever explored the mediating role of cytokines between gut microbiota and high-grade irAEs.

MR uses genetic variants as instrumental variables (IVs) to infer causal links between exposures and outcomes, which helps to minimize confounding factors and the risk of reverse causation and provides more reliable evidence of causality compared to traditional observational studies.^[21–23]^ While next-generation sequencing and third-generation sequencing have unveiled the potential of microbiome-based precision oncology, the mechanistic links between gut microbiota and antitumor immunity remain incompletely understood. Especially in achieving clinical translation, the clinical application of immune therapy optimized by regulating the microbiota is still in its infancy.^[24–26]^ A critical gap lies in identifying the functional mediators that translate microbial signals into systemic immune responses. Based on previous research, we speculate that plasma metabolites and cytokines may precisely be these key intermediates. This study aimed to employ a combined approach of two-sample Mendelian randomization (TSMR) to systematically evaluate the causal effect between gut microbiota (genus *Ruminiclostridium6*) and high-grade irAEs, identify potential significant metabolites and cytokines, and ultimately test whether these metabolites and cytokines play a mediating role.

## 2. Methods

### 2.1. Study design

**Figure 1.**
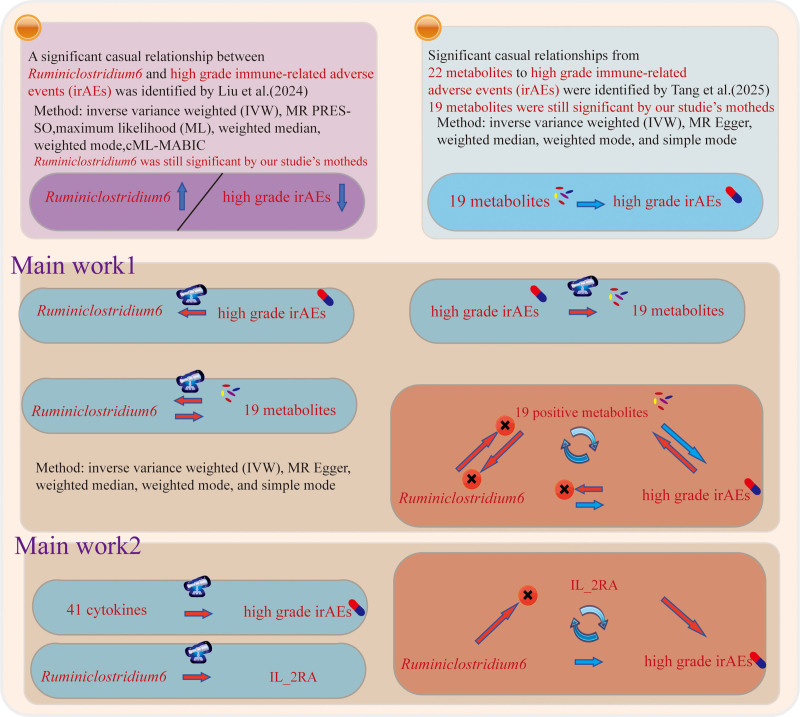
The overall design of the present study.

As shown in Figure 1, the study initially employed a TSMR design to investigate the potential causal relationships between the gut microbiota (genus *Ruminiclostridium6*), high-grade irAEs, and plasma metabolites/cytokines hypothesized to act as a mediating factor. The analysis proceeded in multiple stages. First, genetic instruments variables for plasma metabolites/cytokines, genus *Ruminiclostridium6*, and high-grade irAEs were identified. The bidirectional or unidirectional causal effects between these traits were then assessed using a suite of MR methods to ensure robustness, including inverse variance weighted (IVW), MR Egger, weighted median, weighted mode, and simple mode. Subsequently, a two-step MR approach-based mediation framework was conducted to formally quantify the extent to which the identified significant (positive) plasma metabolites/cytokines mediate such causal effect between *Ruminiclostridium6* and the development of high-grade irAEs. In the meanwhile, sensitivity analyses were processed to assess results of MR analyses and validate their reliability.

### 2.2. Data sources

The genome-wide association studies (GWAS) data of the study conducted by Liu et al^[11]^ was derived from the most comprehensive genome-wide meta-analysis conducted to date by the MiBioGen consortium. However, in the present study, the gut microbiota, named genus *Ruminiclostridium6*, were obtained from the IEU Open GWAS database (https://opengwas.io/) with a OpenGWAS ID of ebi-a-GCST90017050. The data on 19 plasma metabolites were sourced from comprehensive metabolic profiling statistics publicly available in the NHGRI-EBI GWAS Catalog. These measures were obtained from 8299 participants included in The Canadian Longitudinal Study on Aging.^[27]^ Outcome data pertaining to high-grade irAEs were derived from a GWAS conducted by Groha Stefan et al,^[28]^ which focused on cancer patients receiving ICIs. The summary-level statistics for high-grade irAEs are accessible at https://zenodo.org/records/6800429. Additionally, we downloaded the genetic dataset for 41 cytokines from https://data.bris.ac.uk/data/dataset/3g3i5smgghp0s2uvm1doflkx9x.^[29]^ This MR study is a secondary analysis based on publicly available summary-level data from GWAS. All original GWAS included in our analysis have obtained ethical approval from their respective institutional review boards and secured informed consent from all participants. Since our study uses only anonymized, aggregated data, no additional ethics approval was required, and the need for informed consent was waived.

### 2.3. IVs selection

For MR studies, the selection of instruments variables must satisfy the following 3 assumptions to obtain a reliable conclusion: genetic variants must be strongly associated with the exposure; they must not be related to any confounding factors of the exposure–outcome relationship; they must influence the outcome solely through the exposure, not directly or via other pathways.^[30]^ Firstly, we selected the single nucleotide polymorphisms (SNPs) for genus *Ruminiclostridium6* with genome wide-significance (*P*-value < 1 × 10^–5^), corresponding with the study conducted by Liu et al. In terms of 19 plasma metabolites and high-grade irAEs, we also selected a threshold of 1 × 10^–5^ of *P*-value to obtain enough instruments for further analysis. Moreover, a threshold of 5 × 10^–6^ of *P*-value was selected for cytokines to obtain enough instruments. To minimize the effect of linkage disequilibrium, only SNPs meeting the following 2 filter conditions will be involved: the distance between 2 SNPs > 10,000 kb; the *r*^2^ for linkage disequilibrium should <0.001. To exclude the weak IVs for each exposure, *F* statistic >10 were the necessary condition for SNPs to be retained. The *R^2^* and *F* statistic was calculated as the previous studies did.^[31,32]^

### 2.4. Causality analysis between genus *Ruminiclostridium6,* 19 plasma metabolites/41 cytokines and high-grade irAEs

Firstly, we conducted TSMR analyses to explore causal effects of high-grade irAEs on genus *Ruminiclostridium6*. Subsequently, TSMR was conducted to evaluate the causal relationships from high-grade irAEs to 19 plasma metabolites. The bidirectional TSMR was utilized to test the relationships between genus *Ruminiclostridium6* and 19 plasma metabolites. As for 41 cytokines, we firstly evaluated its causal effect on high-grade irAEs to identify significant (positive) cytokines. Then, the causal relationships from *Ruminiclostridium6* to positive cytokines were also detected.

The IVW method or Wald ratio method served as the primary analytical approach, with results presented as *β* and 95% confidence intervals. To complement the IVW estimates, additional methods including MR Egger, simple mode, weighted mode, and weighted median were also employed. In the absence of horizontal pleiotropy, the fixed-effect IVW model provided unbiased causal estimates. In cases where heterogeneity was detected, the random-effect IVW method was adopted to yield more conservative and robust results. When only 1 SNP was available, the Wald ratio method was applied. Subsequently, to functionally interpret the significant metabolites, we performed metabolic pathway enrichment analysis against Kyoto encyclopedia of genes and genomes using the 19 metabolites (MetaboAnalyst 6.0: https://www.metaboanalyst.ca/). Key pathways were identified using an enrichment threshold of *P* < .05.

### 2.5. Mediation analysis

A two-step TSMR framework was utilized to detect mediation pathways. In the aforementioned analysis and previous studies, we validated the significant association between genus *Ruminiclostridium6* and high-grade irAEs, and further assessed the potential causal influence (*β*1) of genus *Ruminiclostridium6* on positive metabolites/cytokines, as well as causal effects (*β*2) from plasma metabolites/cytokines to high-grade irAEs. Should a causal relationship be established, the delta method would be applied to evaluate the significance of the mediating effect and further calculate the proportion of the mediation effect.

### 2.6. Statistical analysis

To evaluate heterogeneity, Cochran *Q* test was implemented based on both IVW and MR-Egger regression. Horizontal pleiotropy was examined using MR-Egger regression and the Mendelian randomization pleiotropy residual sum and outlier framework. Results were visualized using scatter plots, and leave-one-out sensitivity analyses were conducted to assess the influence of individual genetic variants. A two-sided *P*-value of IVW method below .05 were used to initially detect the potential significant causal relationships. Then, to enhance the reliability of findings, only associations with *P*_IVW_ < .05 and consistent effect directions across all 5 MR methods were deemed statistically significant and involved for further analysis. All analyses were performed in R (version 4.4.1) using packages such as“TwoSampleMR.”

## 3. Results

### 3.1. TSMR analysis between 19 plasma metabolites/genus *Ruminiclostridium6* and high-grade irAEs

**Figure 2.**
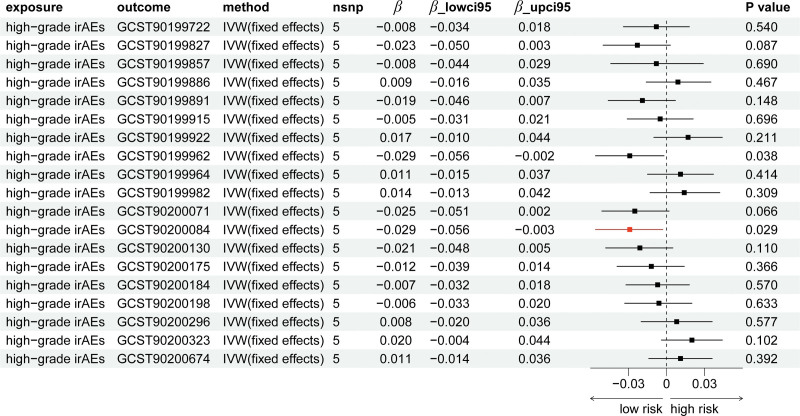
The forest plot of causal effect results by Mendelian randomization of high-grade irAEs on 19 metabolites. irAEs = immune-related adverse events.

**Figure 3.**
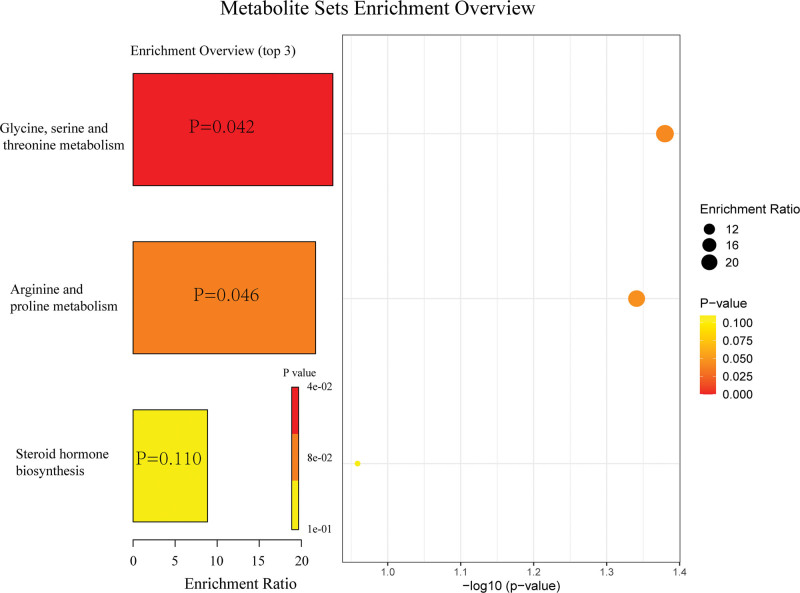
Enrichment analysis results of high-grade irAEs-related plasma metabolites based on KEGG databases. irAEs = immune-related adverse events; KEGG = Kyoto encyclopedia of genes and genomes.

The names of 19 plasma metabolites (positive metabolites) in the present study, significantly associated with high-grade irAEs, can be found in Table S1, Supplemental Digital Content, https://links.lww.com/MD/R489. In the MR analysis from high-grade irAEs to positive metabolites (Table S2, Supplemental Digital Content, https://links.lww.com/MD/R489 and Fig. 2), only Oleoylcholine levels was found to decrease upon the occurrence of high-grade irAEs (*β*_IVW_: ‐0.029). Consistency in the direction of effect estimates was observed across supplementary MR methods (MR-Egger, simple mode, weighted mode, and weighted median), reinforcing the robustness of such association (Table S2, Supplemental Digital Content, https://links.lww.com/MD/R489). Though with *a* < 0.05 *P*_IVW_ value, there lacked a consistency in the direction with other methods for Acisoga levels (Table S2, Supplemental Digital Content, https://links.lww.com/MD/R489 and Fig. 2). As for genus *Ruminiclostridium6*, the direction from high-grade irAEs to genus *Ruminiclostridium6* was not found to own significant causal relationship (Table 1). Scatter plots for the effect of high-grade irAEs on 19 metabolites were shown in Figure S1, Supplemental Digital Content, https://links.lww.com/MD/R488, while the results of leave-one-out analyses can be found in Figure S4, Supplemental Digital Content, https://links.lww.com/MD/R488. The assessment of heterogeneity and horizontal pleiotropy can be found in Tables S3 and S4, Supplemental Digital Content, https://links.lww.com/MD/R489. In addition, based on known metabolites, enrichment analysis indicated that they were significantly (*P* < .05) enriched in the glycine, serine and threonine metabolism and arginine and proline metabolism pathways (Fig. 3). To be noticed, dietary patterns may affect gut microbiota composition, and microbiota changes may in turn influence irAEs risk. However, in the present study, we did not focus on this issue and such analysis was absent.

**Table 1:** The causal effects of high-grade irAEs on genus *Ruminiclostridium6*.

Exposure	Outcome	Method	nSNP	OR (95% CI)	*P* value
High-grade irAEs	ebi-a-GCST90017050	IVW(fixed effects)	5	1.001 (0.981–1.022)	.9052965
High-grade irAEs	ebi-a-GCST90017050	MR Egger	5	0.927 (0.813–1.056)	.3370569
High-grade irAEs	ebi-a-GCST90017050	Simple mode	5	1.001 (0.962–1.041)	.9693385
High-grade irAEs	ebi-a-GCST90017050	Weighted mode	5	1.002 (0.968–1.038)	.9072159
High-grade irAEs	ebi-a-GCST90017050	Weighted median	5	1.003 (0.976–1.031)	.8066085

*Notes*: The *P* value for the heterogeneity analysis is .822 and .682 for MR Egger and inverse variance weighted methods respectively. The *P* value for the pleiotropy analysis is .325. The *P* value for the global test of MR-PRESSO is .690.

CI = confidence interval, irAEs = immune-related adverse events, MR = Mendelian randomization, MR-PRESSO = Mendelian randomization pleiotropy residual sum and outlier.

### 3.2. Bidirectional causal effects between genus *Ruminiclostridium6* and positive metabolites

**Figure 4.**
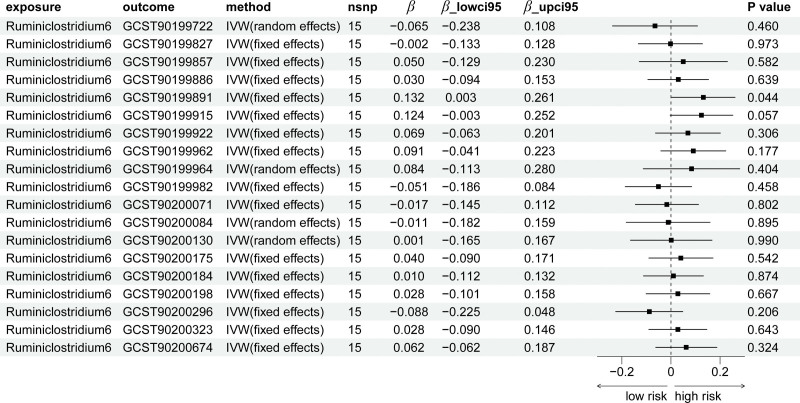
The forest plot of causal effect results by Mendelian randomization of genus *Ruminiclostridium6* on 19 metabolites.

**Figure 5.**
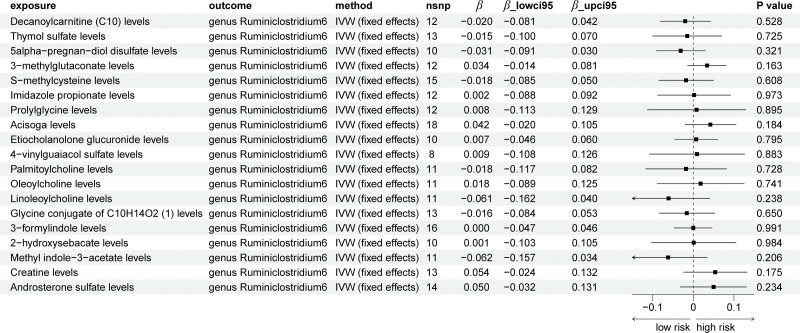
The forest plot of causal effect results by Mendelian randomization of 19 metabolites on genus *Ruminiclostridium6*.

The bidirectional TSMR analysis on genus *Ruminiclostridium6* and positive metabolites can be found in Tables S2 to S4, Supplemental Digital Content, https://links.lww.com/MD/R489. Notably, the analyses did not yield strong evidence for significant causal effects of genus *Ruminiclostridium6* on the positive metabolites at the present study. Though *P*_IVW_ of S-methylcysteine levels <.05, the direction of IVW method is not consistent with other methods (Table S2, Supplemental Digital Content, https://links.lww.com/MD/R489 and Fig. 4). As for reverse analyses, we also found no significant causal relationships between positive metabolites and genus *Ruminiclostridium6* (Table S2, Supplemental Digital Content, https://links.lww.com/MD/R489 and Fig. 5). Scatter plots for the causal effects between genus *Ruminiclostridium6* and 19 positive metabolites were shown in Figures S2 and S3, Supplemental Digital Content, https://links.lww.com/MD/R488. The results of leave-one-out analysis can be found in Figures S5 and S6, Supplemental Digital Content, https://links.lww.com/MD/R488. The assessment of heterogeneity and horizontal pleiotropy can be found in Tables S3 and S4, Supplemental Digital Content, https://links.lww.com/MD/R489.

### 3.3. TSMR analysis associated with cytokines

As shown in Tables S2 to S4, Supplemental Digital Content, https://links.lww.com/MD/R489, only IL_2RA was identified to own significance among the causal relationship (*β*_IVW_ = ‐1.2483, *P*_IVW_ = .049675) from 41 cytokines to high-grade irAEs and no heterogeneity and horizontal pleiotropy were detected. However, IL_2RA lost such significance (Table S2, Supplemental Digital Content, https://links.lww.com/MD/R489) regarding the causal relationship from genus *Ruminiclostridium6* to IL_2RA (*P*_IVW_ = 1.000).

### 3.4. Mediation effects of plasma metabolites/cytokines between genus *Ruminiclostridium6* and high-grade irAEs

Given the causal effect of genus *Ruminiclostridium6* on high-grade irAEs has been validated using the methods in our study,^[11]^ we proceeded to formally test the hypothesis that these metabolites/cytokines act as mediators. A fundamental prerequisite for a meaningful mediation analysis is the existence of a significant causal effect of the exposure (genus *Ruminiclostridium6*) on the proposed mediator (plasma metabolites/cytokines). However, as detailed in sections 3.2 and 3.3, TSMR analyses found limited evidence to support a direct causal influence of genus *Ruminiclostridium6* on the plasma metabolites/cytokines identified as being associated with high-grade irAEs. The associations observed were either nonsignificant or demonstrating inconsistent effects across MR methods, failing to provide a robust foundation for mediation. Consequently, these specific plasma metabolites do not function as mediators in the causal relationship between genus *Ruminiclostridium6* and the development of high-grade irAEs. Similarly, as for cytokines, though a causal relationship between IL_2RA and high-grade irAEs was identified, the mediating role still cannot be established for the absence of its significant causal relationship with genus *Ruminiclostridium6.*

### 3.5. Sensitivity analyses

To assess the robustness of the causal estimates derived from the MR analyses, we conducted comprehensive sensitivity analyses, including tests for heterogeneity and pleiotropy, as well as leave-one-out validation. Heterogeneity was evaluated using Cochran *Q* statistic with MR-Egger and IVW methods, as summarized in Table S3, Supplemental Digital Content, https://links.lww.com/MD/R489. To examine the potential influence of horizontal pleiotropy, we mainly relied on the MR-Egger intercept test supplemented by the Mendelian randomization pleiotropy residual sum and outlier global test, which is presented in Table S4, Supplemental Digital Content, https://links.lww.com/MD/R489. Additionally, leave-one-out sensitivity analyses were performed by iteratively excluding each SNP and recalculating the MR estimates.

## 4. Discussion

### 4.1. Where we are and where we’re going

To date, several MR-based studies have successfully established causal roles for various factors in irAEs pathogenesis, including specific gut microbiota taxa,^[11]^ plasma proteins (e.g., CCL20, CXCL9),^[33]^ immune cell characteristics,^[34]^ and clinical risk factors such as BMI and autoimmune diseases.^[35]^ There were several studies having explored the association between the metabolites and irAEs. For example, Gao et al^[4]^ demonstrated that specific metabolic profiles, including acylcarnitines, were significantly different between the irAE and non-irAEs groups, which highlights an important role of the metabolites for the development of irAEs. Gao et al^[4]^ discussed alterations of microbial metabolites that have been associated with irAEs, and their potential mechanistic links. Moreover, recently, Zhu et al^[9]^ identified signatures of metabolites in patients with severe irAEs compared to those in patients experiencing mild or no irAEs in a cohort of 168 patients. Tang et al^[18]^ elucidated the role of metabolites in the causal effect of immune cell phenotypes on the immunotherapy toxicity risk.

In terms of cytokines, many cytokines have been demonstrated to be associated with high-grade irAEs, including IL-7, IL-1RA, CXCL-13, IL-16, IL-12, and so on.^[19,20,36]^ Up to now, cytokine pathway inhibitors, particularly anti-TNFa and anti-IL-6R antibodies, are commonly used as second-line immunosuppression. In a review from Hadfield et al, they also discussed other cytokines implicated in irAE pathophysiology, such interleukin-17 (IL-17) and interleukin-4/13 (IL-4/IL-13).^[37]^ However, there still laid a gap for how the metabolites and cytokines would affect and mediate the “gut microbiota-associated” development of the severe irAEs. By performing a comprehensive MR investigation and explicitly testing the mediation of the gut microbiota-high-grade irAEs relationship by a broad plasma metabolome/cytokines panel, our study contributed to the evolving landscape of MR applications in such gap.

### 4.2. Summary of the finding of the present study

In the present study, we investigated the causal relationships between 19 positive metabolites/41 cytokines, the gut microbial genus *Ruminiclostridium6*, and the risk of high-grade irAEs. Our analyses yielded the following key findings: the high-grade irAEs may not have a genetic influence on the abundance of genus *Ruminiclostridium6.* Reverse MR analysis indicated that the occurrence of high-grade irAEs might lead to decreased levels of oleoylcholine. Bidirectional TSMR analysis between genus *Ruminiclostridium6* and the 19 irAE-associated plasma metabolites did not provide robust evidence for significant causal relationships in either direction. A significant causal relationship was identified from IL_2RA to high-grade irAEs. Based on a framework of two-step TSMR, the 19 positive metabolites and 1 positive cytokines (IL_2RA) did not appear to mediate the causal relationship between genus *Ruminiclostridium6* and high-grade irAEs.

Additionally, in the metabolite enrichment analysis, we identified the glycine, serine and threonine metabolism and arginine and proline metabolism pathways may influence the development of the high-grade irAEs. This provided a potential target for further researches to explore the ways to inhibit the occurrence of the high-grade irAEs. This provides a potential target for further research to explore therapeutic strategies for mitigating severe irAEs. Specifically, modulation of this metabolic pathway could offer a novel approach to prevent or reduce the incidence of these adverse events, potentially through dietary interventions, pharmacological inhibitors, or targeted metabolic reprogramming. Future studies should validate these findings in preclinical models and elucidate the precise mechanistic links between dysregulated amino acid metabolism and immune-related toxicity.

### 4.3. Possible explanation to the non-mediating role of metabolites and cytokines

Our mediation analysis revealed no significant mediating effect of the identified significant high-grade irAEs-related plasma metabolites/cytokines from genus *Ruminiclostridium6* to high-grade irAEs. The lack of observed mediation suggests several possible mechanistic explanations. First, the protective effect of genus *Ruminiclostridium6* on the severity of irAEs may operate through local gut-level mechanisms rather than the changes of metabolites/cytokines detectable in plasma. Previous research has highlighted the importance of gut microbiota in modulating mucosal immunity and maintaining intestinal barrier function, which could influence irAEs development through direct immune cell interactions rather than circulating metabolites.^[10,38]^ Secondly, the mediating factors might involve microbial-derived metabolites not captured by the 1400-metabolite and 41-cytokine panel used in this study, such as short-chain fatty acids, bile acid derivatives, or other specialized microbial compounds that exert local immunomodulatory effects. Actually, several studies have emphasized the role of specific microbial metabolites in immunotherapy response, suggesting that more targeted metabolic profiling might be necessary.^[39,40]^ Third, the causal pathway might involve more complex circuits incorporating immune cell populations as intermediaries.^[18]^ For instance, Shi et al^[34]^ demonstrated that immune cell characteristics mediate the relationship between plasma metabolites and irAEs, suggesting that the gut microbiota-high grade irAEs relationship might be routed through immune cell modulation rather than directly through plasma metabolites.

### 4.4. Limitations

Several limitations should be considered when interpreting our results. Firstly, the plasma metabolome data, while extensive, may not capture all relevant metabolite classes, particularly those derived specifically from gut microbial metabolism. Secondly, the European ancestry of the GWAS data limits the generalizability of our findings to other populations. Thirdly, due to the utilization of summary statistics of the irAEs GWAS data, subgroup analyses for different cancer types and different regimes are restricted. Thirdly, the mediation analysis rests on the assumption of a linear causal pathway from gut microbiota to metabolites/cytokines to irAEs. However, the relationship may be more complex, involving nonlinear dynamics, tissue-specific metabolic effects, or intermediary roles of immune cell populations not captured in the present study. Additionally, further studies are needed to elucidate how distinct microbial communities at various barrier sites (e.g., the gut and other mucosal surfaces) influence the development of irAEs. Finally, external factors (such as diet, environment, or life style) may influence gut microbiota composition; and microbiota changes may in turn influence irAEs risk, possibly confounding the results of our analysis.^[32]^

## 5. Conclusion

In summary, this MR study found no evidence that the protective association between genus Ruminiclostridium6 and high-grade irAEs was mediated through the circulating levels of the 19 irAE-associated metabolite/41 cytokines. While a causal effect of high-grade irAEs on lowering oleoylcholine was identified, and IL-2RA was linked to high-grade irAE risk, these factors did not serve as intermediates in the microbial pathway. However, additional randomized clinical trials are needed to determine whether fecal microbiota transplantation or probiotics can mitigate irAEs while preserving the efficacy of ICIs.

## Acknowledgments

We would like to thank Zhenli Li who helped us to make necessary data analysis.

## Author contributions

**Conceptualization:** Sihan Liu, Zhenli Li.

**Data curation:** Sihan Liu, Jingtao Ma, Zhenli Li.

**Formal analysis:** Sihan Liu, Zhenli Li.

**Validation:** Zhenli Li.

**Writing – original draft:** Sihan Liu.

**Writing – review & editing:** Jingtao Ma, Mengjia Li, Tenghui Wang, Tiezhu Yao, Guang Liu.

## Supplementary Material




