# Supplementary material for: Explore the potential mediating role of plasma metabolites and cytokines in the causal relationship between gut microbiota and the severity of immune-related adverse events: A Mendelian randomization study
Source: Medicine (Baltimore). 2026 Feb 28;105(9):e47916. doi: 10.1097/MD.0000000000047916 (PMC12956236; doi:10.1097/MD.0000000000047916)

Additional Figures

Figure S1. Scatter plots for the effect of high-grade irAEs on 19 metabolites. irAEs= immune-related adverse events.

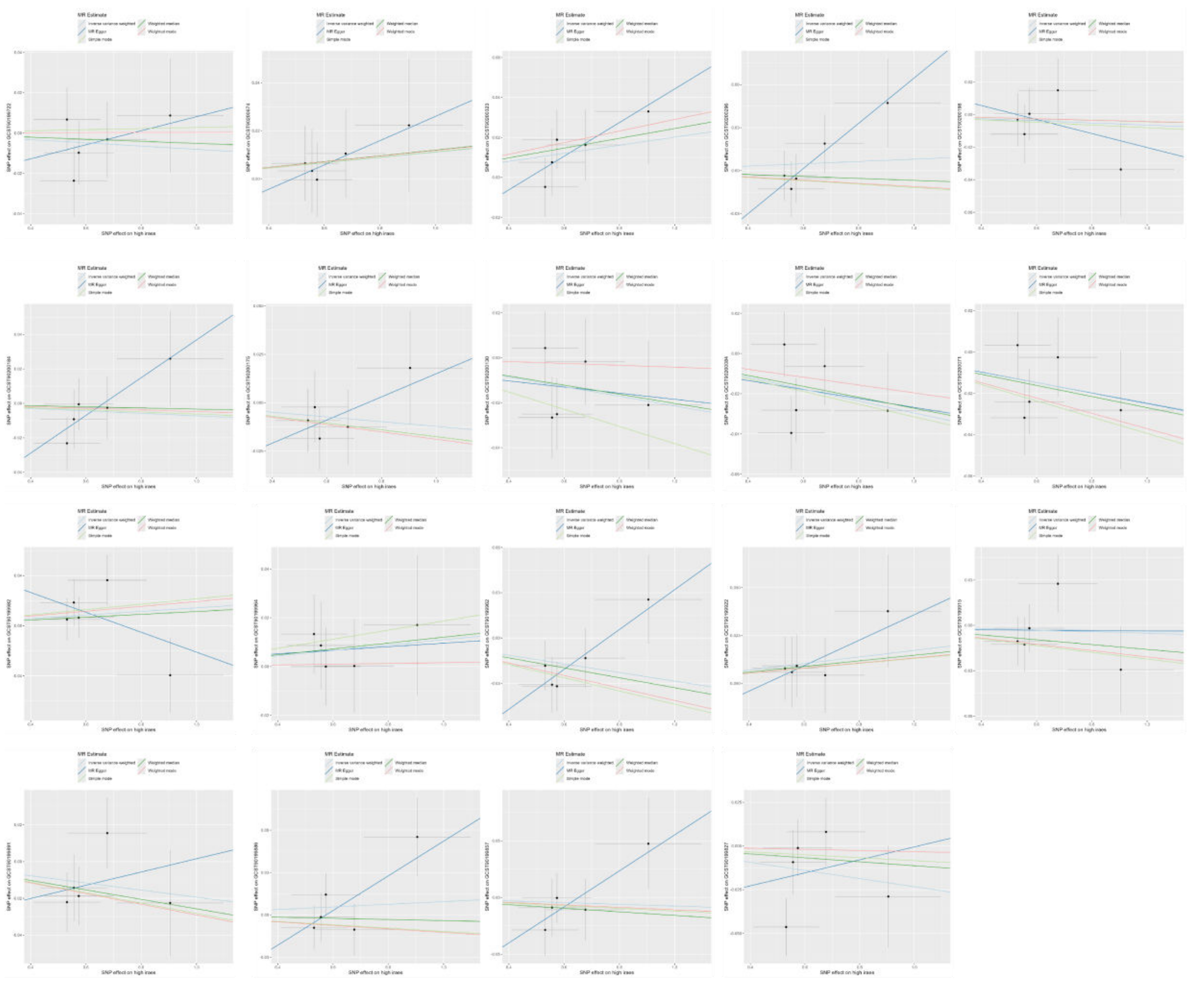

Figure S2. Scatter plots for the effect of genus *Ruminiclostridium6* on positive metabolites.

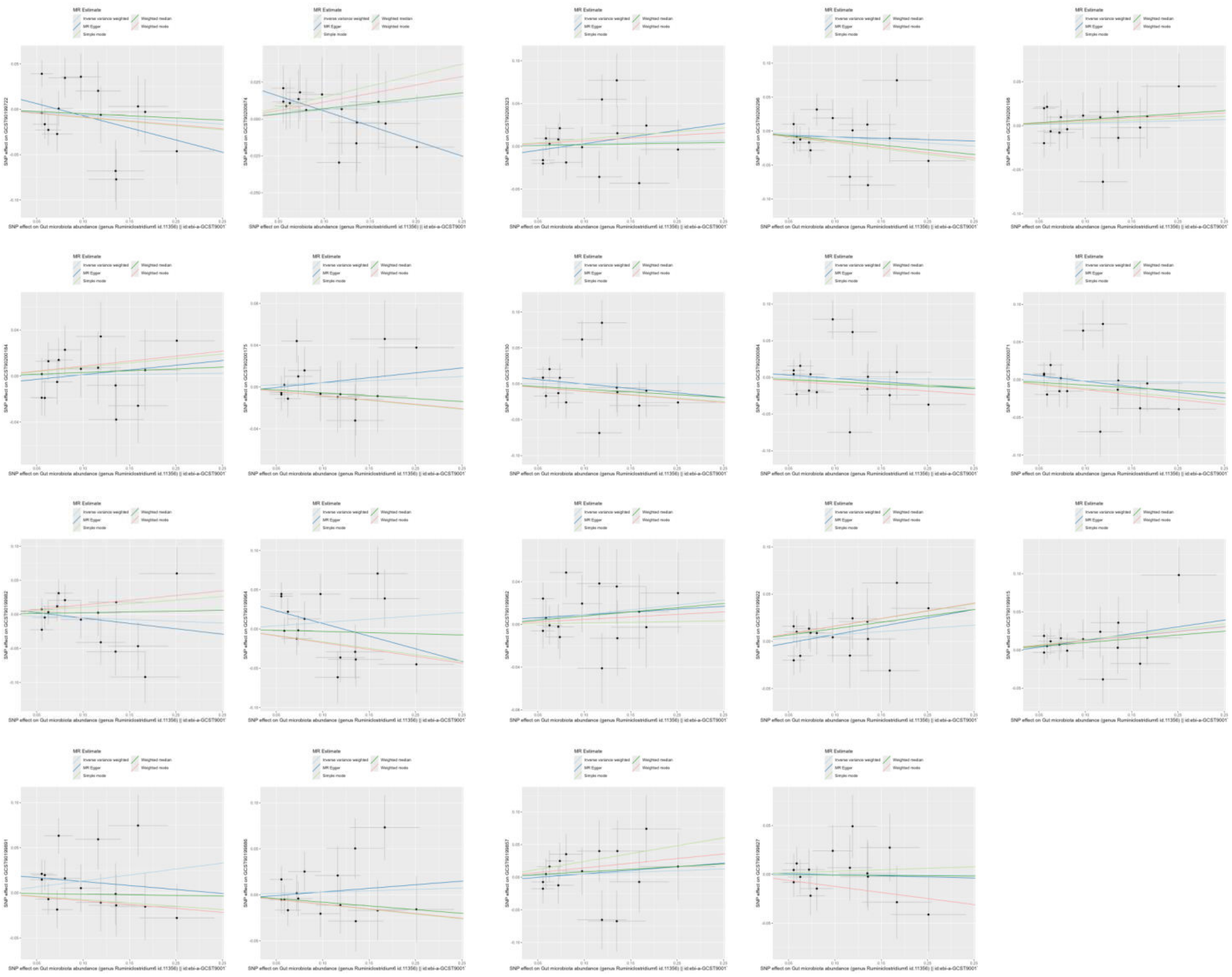

Figure S3 Scatter plots for the effect of positive metabolites on genus *Ruminiclostridium6* .

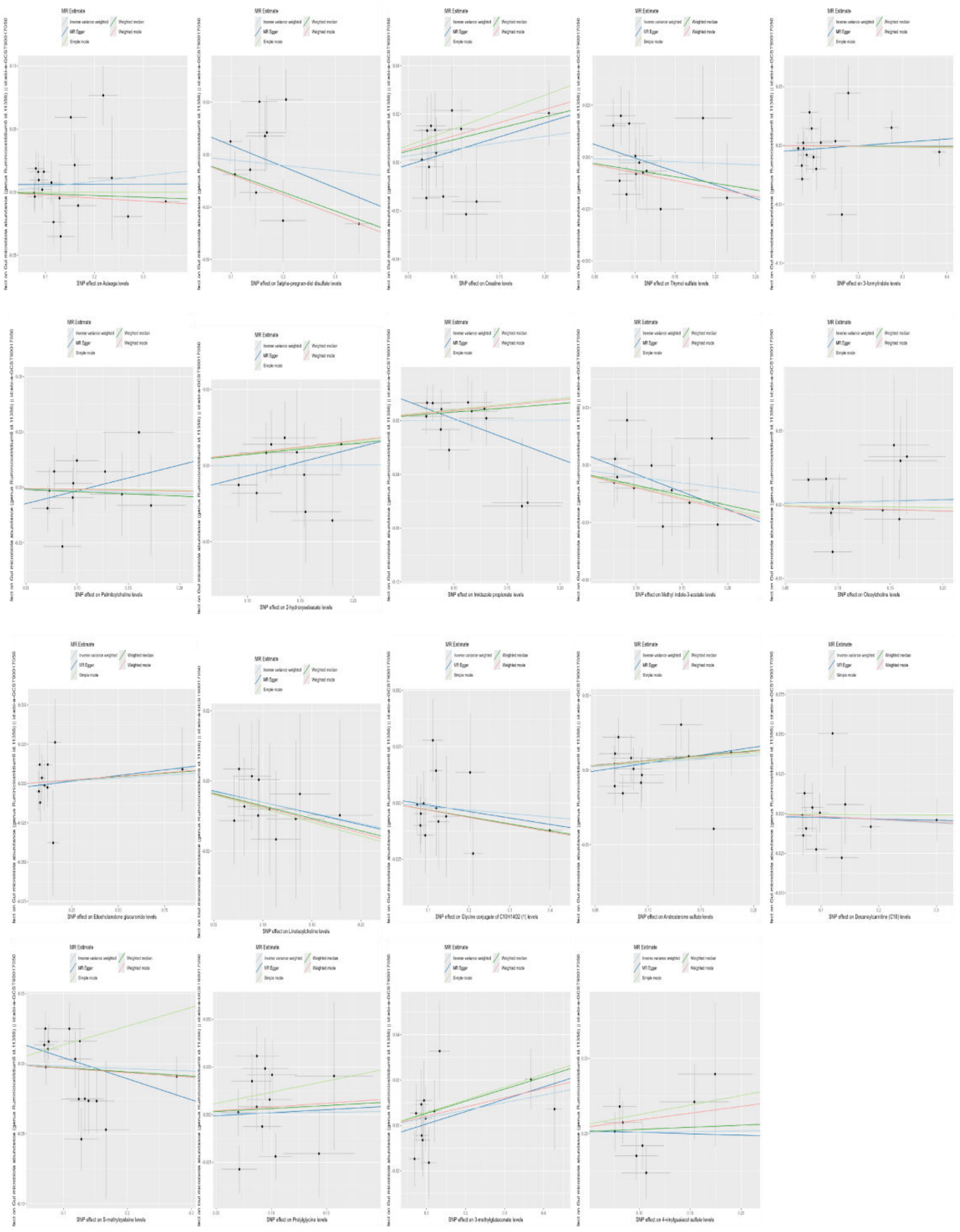

Figure S4. MR leave-one-out sensitivity analysis for the effect of high grade irAEs on 19 metabolites. irAEs= immune-related adverse events.

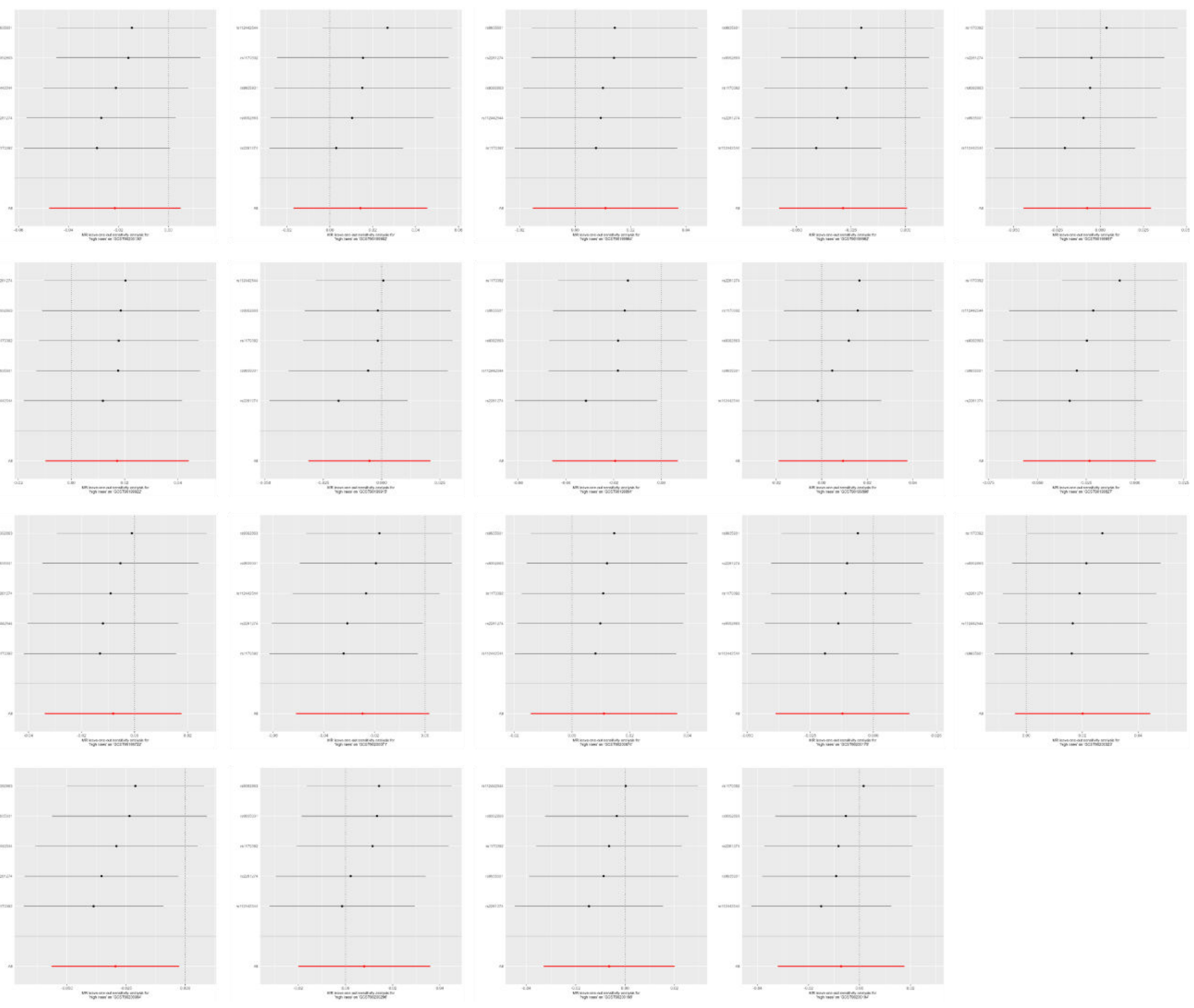

Figure S5. MR leave-one-out sensitivity analysis for the effect of genus *Ruminiclostridium6* on positive metabolites.

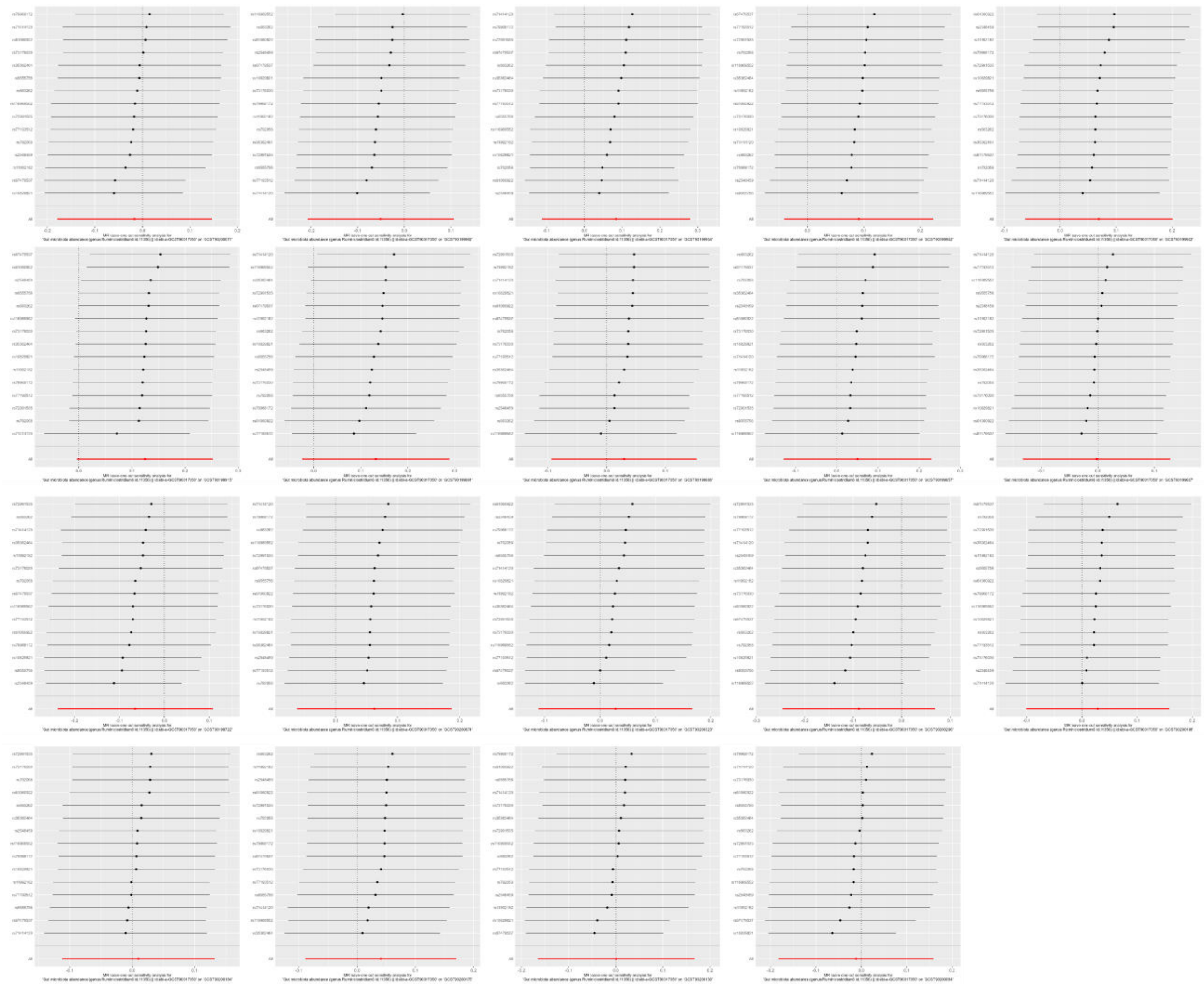

Figure S6. MR leave-one-out sensitivity analysis for the effect of positive metabolites on genus *Ruminiclostridium6*.

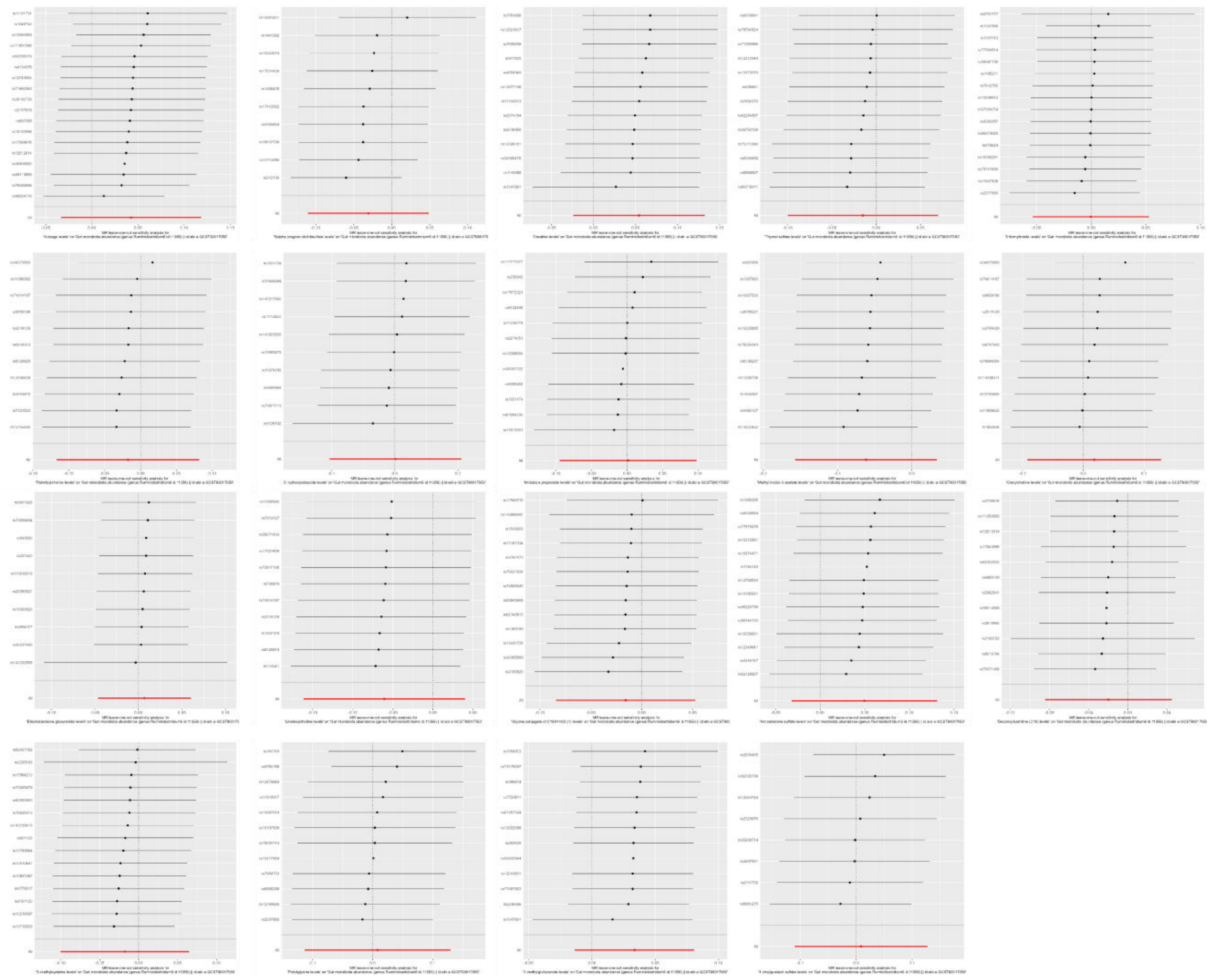

Supplement: Supplementary file 2 [file medi-105-e47916-s002.pdf]
